# Genetic heterogeneity affects the risk of incident depression, comorbidity, and response to environment: A prospective trajectory study

**DOI:** 10.1017/S0033291726104140

**Published:** 2026-06-22

**Authors:** Chuyu Pan, Shiqiang Cheng, Xin Qi, Bolun Cheng, Jin Feng, Meijuan Kang, Li Liu, Xuena Yang, Yan Wen, Yumeng Jia, Huan Liu, Feng Zhang

**Affiliations:** 1 Key Laboratory of Trace Elements and Endemic Diseases of National Health and Family Planning Commission, Key Laboratory of Environment and Genes Related to Diseases of Ministry of Education of China, Key Laboratory for Disease Prevention and Control and Health Promotion of Shaanxi Province, School of Public Health, Health Science Center, Xi’an Jiaotong University, Xi’an, China; 2 Precision Medicine Center, The First Affiliated Hospital of Xi’an Jiaotong University, Xi’an, P. R. China

**Keywords:** comorbidity, depression, environment, genetics, heterogeneity, trajectory

## Abstract

**Background:**

Depression exhibits significant heterogeneity in its genetic underpinnings. The role of genetic components in the development of depression and its comorbidities remains insufficiently explored.

**Methods:**

First, depression risk loci from a large-scale genome-wide meta-analysis were annotated to Gene Ontology (GO) terms by functional enrichment. GO-based polygenic risk scores (GO-PRS) were then calculated for individuals in the UK Biobank. Principal component analysis (PCA) was applied for dimensionality reduction, followed by cluster analysis to identify genetic subtypes of depression. Multistate models were applied to assess the impact of genetic patterns on the trajectory from healthy status to incident depression, and depression to 26 subsequent diseases, as well as the associations between environmental factors and disease trajectories across genetic subtypes.

**Results:**

Participants were categorized into three genetic subtypes: immune-dominant, neuro-dominant, and comprehensive-risk. Significant differences in risk of depression and subsequent diseases, and susceptibility to environmental factors were observed across subtypes. Comprehensive-risk subtype showed higher risks of depression compared to immune-dominant (HR: 1.10, 95% CI: 1.05–1.15) and neuro-dominant subtype (HR: 1.12, 95% CI: 1.08–1.16). Comprehensive-risk subtype exhibited higher risks of transition from depression to subsequent diseases, such as anemia compared to immune-dominant subtype, and diseases of the digestive system compared to neuro-dominant subtype. Environmental factors were more strongly associated with the transition from depression to subsequent diseases in immune-dominant and comprehensive-risk subtypes, including cardiovascular, respiratory, and metabolic diseases.

**Conclusions:**

Our findings highlight the genetic heterogeneity of depression and comorbidities, and shed light on how genetic components modulate responses to environmental factors.

## Introduction

Depression is a major global health issue and one of the leading causes of disability worldwide (GBD 2019 Mental Disorders Collaborators, 2022). Its prevalence has been steadily increasing, affecting approximately 300 million people globally (Nagy et al., 2020). In addition to its direct impact on mental health, depression is also associated with an increased risk of developing various physical illnesses, including cardiovascular diseases, metabolic and endocrine disorders, neurodegenerative diseases, and autoimmune diseases (Frank et al., 2023). This extensive comorbidity amplifies the overall disease burden, leading to poorer prognoses (Bai et al., 2021), increased mortality rates (Stubbs et al., 2017; Weye et al., 2020), and substantially higher healthcare utilization and costs (Christensen et al., 2023). Identifying individuals at high risk and modifiable risk factors that contribute to the progression of depression to other chronic diseases is crucial for alleviating the burden of depression.

Depression exhibits considerable individual variability in risk factors, genetic background, and comorbidities (Felger & Miller, 2020; Fried & Nesse, 2015). This heterogeneity is a key challenge in the diagnosis and treatment of depression (Fried & Nesse, 2015). Currently, the role of various genetic components in the development of depression and its comorbidities remains underexplored. Although a recent study identified genetic characteristics predominantly linked to the immune system in the context of age-dependent multi-morbidities across depression subtypes (Nguyen et al., 2023), the biological processes enriched by genetic loci in genome-wide association studies (GWAS) have extended beyond immune-related pathways, encompassing synaptic structure and function, neuronal development, neurotransmission, and stimulus response (Howard et al., 2019). Considering genetic heterogeneity, we hypothesize that genetic predispositions to depression may derive their risk from different biological processes among individuals. In some cases, the primary genetic risk may be concentrated in loci associated with neural functions, potentially affecting synaptic structure, neuronal development, or neurotransmission (Garvert, Kirchner, Grabe, & Van der Auwera, 2022; Wainberg, Jacobs, Voineskos, & Tripathy, 2022). In others, the risk may predominantly arise from loci regulating inflammation and immune responses (Gezsi et al., 2024; Shen et al., 2022). Alternatively, genetic risk may reflect a combination of multiple risk-related pathways.

Moreover, interactions between genetic factors and environmental exposures are thought to play an important role in depression susceptibility, with variations in genetic architecture shaping individual responses to environmental risk factors (Cooper, 2003). Genetic predispositions associated with distinct biological mechanisms may exhibit varying vulnerabilities and responses to specific environmental exposures. Although prior research has linked the mixed atypical-melancholic subtype of depression to low levels of physical activity among various subtypes (Rovero et al., 2024), the integration of genetic subtypes with environmental interactions remains limited. Previous investigations on the environmental factors for depression and its comorbidities have overlooked genetic heterogeneity, which may reduce statistical power and obscure true associations. Clarifying how environmental factors influence the risk of depression comorbidities in individuals with distinct genetic components could enhance our understanding of disease mechanisms. It may also help identify high-risk individuals who are exposed to specific risk factors and inform targeted prevention strategies.

Genetic risk for complex diseases can be partitioned into biologically meaningful modules to facilitate the investigation of etiological heterogeneity (Choi et al., 2023). In this study, we classified depression-related genetic loci into distinct biological functional modules; each module represents a unique set of biological processes that may contribute to the development of depression. Then, we assessed the polygenic risk of these modules to identify specific genetic components and stratify individuals into distinct genetic subtypes. We reasoned that, if the identified genetic subtypes capture biologically meaningful heterogeneity, they would be expected to demonstrate distinct risks of incident depression, differential trajectories from depression to subsequent diseases, and heterogeneous associations with environmental exposures. Through this approach, we aimed to gain deeper insights into the genetic heterogeneity of depression, its implications for depression and subsequent chronic disease risk, and the extent to which genetic subtypes modify the associations between environmental exposures and the risks of depression and its comorbid conditions.

## Methods

### Participants and study design

This prospective study was conducted based on the UK Biobank, a large-scale population-based prospective study that contains biological samples and phenotype data from over 500,000 people aged 40–69 years assessed between 2006 and 2010. This study encompasses 22 assessment centers across the United Kingdom. During this period, participants completed self-administered touch-screen questionnaires and underwent brief computer-assisted interviews as part of their assessment visit. Ethical approval for the UK Biobank study was obtained from the North West Multi-center Research Ethics Committee, under approval number 11/NW/0382. All participants provided informed consent, granting UK Biobank access to their health-related records (Sudlow et al., 2015).

**Figure 1.**
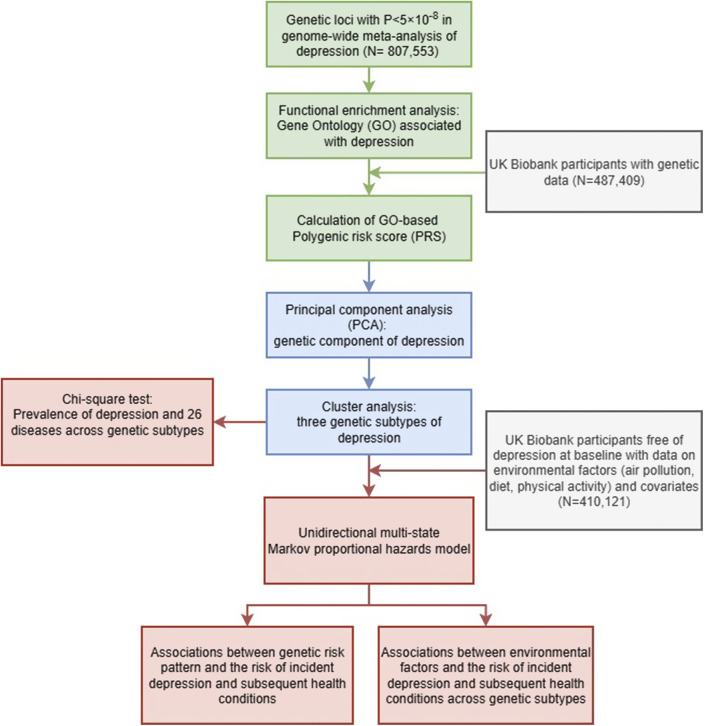
Flow chart of the study. Figure 1. long description.

In this study, the genetic loci from GWAS of depression were first annotated to Gene Ontology (GO) terms through functional enrichment analysis. A GO-based polygenic risk score (GO-PRS) was calculated for each GO term using its corresponding depression-associated loci among 487,409 UK Biobank participants with available genotype data. These scores were then subjected to principal component analysis (PCA) to identify underlying genetic characteristics. Cluster analysis was performed to group individuals with different genetic characteristics into different clusters. Finally, Cox proportional hazards regression and multi-state models were used to assess the impact of those genetic patterns on the risk of depression and the trajectory from depression to subsequent diseases, as well as the impact of environmental factors on the risk of depression and disease trajectories across different genetic subtypes (Figure 1).

### Environmental factors

According to previous studies, diet, air pollution, and physical activity were reported to be associated with the risk of depression (Gao et al., 2023; Marx et al., 2021; Pearce et al., 2022). However, it remains unclear how these environmental factors relate to depression across different genetic subtypes. Therefore, we investigated the effects of these factors on the risk of depression and its comorbidities within distinct genetic subtypes. The detailed definition is shown in the Supplementary Materials.

The air pollution data were derived from the European Study of Cohorts for Air Pollution Effects (ESCAPE) project, covering 20 European regions for particulate matter research and 32 regions for nitrogen oxide research (Beelen et al., 2013; Eeftens et al., 2012). Land-use regression models estimated participants’ residential exposure based on home addresses (Beelen et al., 2013; Eeftens et al., 2012). Specifically in this study, nitrogen dioxide (NO_2_), PM_2.5_ particulate matter with aerodynamic diameter ≤ 2.5 μm (PM_2.5_), and particulate matter with aerodynamic diameter > 2.5 μm and ≤ 10 μm (PM_2.5–10_) were used as primary air pollution indicators. The exposure levels for NO_2,_ PM_2.5_, and PM_2.5–10_ were determined using the 2010 annual average concentrations.

Diet was assessed using a healthy diet score based on the Mediterranean diet and heart-healthy dietary recommendations for reducing the risk of chronic diseases (Lourida et al., 2019; Mozaffarian, 2016), including seven components: fruits, vegetables, fish, processed meat, unprocessed red meat, whole grains, and refined grains. The healthy diet score was calculated by summing the scores for each of the seven food components consumed by each participant, with a range from 0 to 7 (Lourida et al., 2019). The healthy diet scores were classified into three categories: low (0–1 points), moderate (2–5 points), and high (6–7 points) diet scores (Schulz et al., 2023).

Physical activity was evaluated using the International Physical Activity Questionnaire (IPAQ), and participants were classified into three activity levels: high, moderate, and low (Sjostrom et al., 2005). The high IPAQ group engaged in ≥1 hour of moderate- or ≥ 30 minutes of vigorous-intensity activity daily above basal levels. The moderate group was defined as doing some activity, roughly equivalent to 30 minutes of moderate-intensity physical activity on most days. The low IPAQ group included those not meeting either criterion (Sjostrom et al., 2005).

### Outcomes and follow-up

During the follow-up period, the occurrences of diseases were defined according to the ICD-10. Depression was defined based on ICD-10 codes F32-F33. A total of 26 diseases were considered in this study, including metabolic, cardiovascular, respiratory, digestive, neurological, musculoskeletal, hematological, infectious, psychiatric, renal, and sensory system disorders. The baseline was set as the date of attending the assessment center. The follow-up time was calculated from the baseline date until the diagnosis of the disease outcome, death, loss to follow-up, or the end of the follow-up on December 31, 2019, whichever occurred first. Participants who were diagnosed with the disease before the deadline were coded as 1, while those who did not experience the disease or death or were lost to follow-up before the deadline were coded as 0. Individuals diagnosed with the corresponding disease at baseline were excluded. Detailed definitions of diseases are provided in the Supplementary Materials.

### Functional annotation of depression related genetic loci

Depression-related genetic loci were derived from a genome-wide meta-analysis combining data from 33 cohorts of the Psychiatric Genomics Consortium including 500,199 individuals (170,756 cases and 329,443 controls) (Howard et al., 2019). Meta-analysis was performed using Metal software (Willer, Li, & Abecasis 2010), conditioned on the presence of each variant in the studies. Linkage disequilibrium score (LDSC) regression intercepts were used for genomic inflation control of contributing cohorts and the final meta-analysis results. The detailed protocols of GWAS meta-analysis can be found in previous studies (Howard et al., 2018; Howard et al., 2019; Wray et al., 2018).

Depression-related loci with *P* < 5 × 10^−8^ were mapped to genes using the Varnote tool (http://www.mulinlab.org/varnote/application.html#REG) and subsequently subjected to functional enrichment analysis (D. Huang et al., 2020). Functional enrichment analysis was conducted to identify GO terms associated with depression-related genes, using the Functional Mapping and Annotation (FUMA) software, an integrative web-based platform that leverages multiple biological resources to facilitate functional annotation in a biological context (Watanabe, Taskesen, van Bochoven, & Posthuma, 2017). The overrepresentation of biological functions of prioritized genes was tested against gene sets obtained from MsigDB and WikiPathways by hypergeometric tests (Watanabe, Taskesen, van Bochoven, & Posthuma, 2017). Finally, 4,625 depression-related loci were mapped to 209 genes and included in the analysis. The Benjamini–Hochberg correction was applied to each data source of tested gene sets.

### GO-PRS

PRS for GO terms were calculated using PLINK 2.0 among 487,409 genotyped participants from the UK Biobank (Bani-Fatemi et al., 2019; Chang et al., 2015; Liyanage et al., 2022). To account for linkage disequilibrium (LD), a clumping procedure was applied to retain approximately independent SNPs (Bani-Fatemi et al., 2019; Chang et al., 2015; Liyanage et al., 2022). For each GO term, the SNPs with *P* < 5 × 10^−8^ were clumped using a window size of 250 kb and a threshold of r^2^ < 0.2 (Pan et al., 2024; Sikdar et al., 2021). The pairwise LD (r^2^) values were calculated based on maximum likelihood estimates of haplotype frequencies. The formula for GO-PRS calculation is as follows:

$$PRS=\sum_{i=1}^{j}b_{i}d_{i}$$

For each individual, i ( i =1,2,3… j ) represents the number of SNPs enriched in GO; $b_{i}$ is the effect parameter of depression for the risk allele of the i -th SNP, and $d_{i}$ is the dose (0 to 2) of the risk allele for the i -th SNP.

### PCA

PCA has been widely applied in reducing the dimensionality of the genetic data while retaining most of the variation in the data set (Ringnér, 2008). In the context of genome-wide data, several studies have employed PCA to identify complex genetic structures, especially for identifying population stratification or latent genetic factors (Turiaco, Iannuzzo, Bruno, & Drago, 2024). In this study, PCA was used to transform the original set of GO-PRS into a new set of orthogonal components, which may capture the genetic components of depression. The original PRS was standardized to eliminate the impact of different variable scales on the analysis results. The analysis was conducted on 39 GO-PRS variables, resulting in 39 principal components, ordered by the proportion of variance they explained. To achieve dimensionality reduction while retaining the majority of the dataset’s informational content, principal components accounting for 90% of the cumulative variance were retained (Jolliffe & Cadima, 2016; Pillinger et al., 2023). The selected components were then used for subsequent clustering analyses. PCA was conducted using ‘prcomp’ function in R 4.4.0.

### Cluster analysis

*K*-means clustering was performed on the principal components derived from PCA to partition the samples into *k* distinct clusters based on their genetic characteristics. The objective of clustering was to group samples with similar genetic patterns while maximizing the differences between clusters. To determine the optimal number of clusters, the total within-cluster sum of squares (WSS) was calculated for various values of *k*. The ‘elbow’ method was employed by plotting the WSS against different values of *k*, and the optimal number of clusters was identified at the point where the curve exhibited an inflection, known as the ‘elbow’. This method allowed for the identification of clusters representing distinct genetic patterns within the population (Bock, 2008). Cluster analysis was performed using ‘cluster’ package in R 4.4.0.

Clustering quality was assessed using the entropy index and pairwise Mahalanobis distances. The entropy index was calculated based on posterior cluster membership probabilities to quantify classification uncertainty. Pairwise Mahalanobis distances between clusters were computed using cluster centroids and the pooled covariance matrix to evaluate multivariate separation among subtypes.

### Statistical analysis

The mean and standard deviation (SD) were used to describe continuous variables with normal distribution, and numbers (percentages) were used to describe categorical variables. A chi-square test was used to assess differences in the prevalence of depression, other 26 diseases, and comorbid conditions across individuals with different subtypes. P-values were adjusted for multiple comparisons using the Bonferroni correction.

A multistate Markov proportional hazards model was employed to examine the impact of genetic patterns on the risk of depression and the risk of transition from depression to chronic disease, and the influence of environmental exposures on the risk of depression and progression trajectories from incident depression to chronic diseases among individuals with distinct genetic patterns. Multistate models, as probabilistic frameworks, encompass multiple states and allow modeling of transition rates between these states (de Wreede, Fiocco, & Putter, 2010; H. Huang et al., 2026; Luo et al., 2022). This approach ensures that only individuals who are free of the target event at the start of each transition contribute to the corresponding risk set. Two transitions were defined in the multistate model: transition 1: baseline health to depression; transition 2: depression to subsequent chronic diseases. The ‘mstate’ version 0.3.2 R package was used to implement the multistate models (de Wreede, Fiocco, & Putter, 2010). Basic demographic variables (gender, age), socioeconomic indicators (education level, social deprivation, household income), and lifestyle factors (smoking and drinking status) were considered covariates. When exposure involved air pollution, local environmental exposures (24-hour weighted average noise and distance to major roads) were also considered covariates. To ensure that exposure estimates reflected meaningful long-term exposure relevant to disease risk, we excluded participants who had resided at their current address for less than five years. We further conducted sensitivity analysis excluding road proximity and average noise level as covariates. The detailed definition of covariates can be seen in the Supplementary Materials.

Moreover, Spearman correlation analyses were conducted to provide scale-free comparisons of correlation patterns. Spearman rank correlation coefficients were first calculated to assess associations between subtype and subsequent diseases after depression, as well as between environmental exposures and diseases within each genetic subtype. To compare correlation strength, nonparametric bootstrap resampling with replacement (500 iterations) was then applied. Within each bootstrap sample, Spearman correlations were recalculated, and pairwise differences in correlation coefficients were obtained. Ninety-five percent confidence intervals (95% CI) were derived from the bootstrap distributions, and differences were considered statistically significant if the confidence interval did not include zero.

## Results

### Basic characteristics of participants

In total, 410,121 participants (mean [SD] age, 56.17 [8.09]) were included in the analysis, with 195,716 (47.7%) being male. During a median (IQR) follow-up of 10.8 (10.1–11.5) years, 17,072 individuals developed depression. The demographic characteristics of participants are shown in Table 1.

**Table 1.** Demographic characteristics of participants Table 1. long description.

	N = 410,121	
	Control (N = 393,049)	Depression (N = 17,072)
Sex, male (%)	189,344 (48.2)	6,372 (37.3)
Age (%)		
≥65 years old	69,549 (17.7)	3,041 (17.8)
<65 years old	323,500 (82.3)	14,031 (82.2)
TDI (mean [SD])	−1.40 (3.03)	−0.63 (3.32)
Education attainment (%)		
University or college degree	141,138 (35.9)	4,528 (26.5)
Other	251,911 (64.1)	12,544 (73.5)
Household income (%)		
Low	185,297 (47.1)	11,114 (65.1)
Moderate	103,789 (26.4)	3,619 (21.2)
High	103,963 (26.5)	2,339 (13.7)
Ever drank alcohol, yes (%)	378,256 (96.2)	16,256 (95.2)
Ever smoked, yes (%)	177,229 (45.1)	9,336 (54.7)
Cluster (%)		
1	156,928 (39.9)	6,489 (38.0)
2	75,209 (19.1)	3,184 (18.7)
3	160,912 (40.9)	7,399 (43.3)
NO_2_, high (%)	189,381 (48.9)	8,939 (53.0)
PM_2.5,_ High (%)	176,816 (49.3)	9,153 (54.9)
PM_2.5–10_, High (%)	177,763 (49.5)	8,349 (50.1)
Distance to major roads, 1/km (mean [SD])	0.01 (0.02)	0.01 (0.01)
24-hour weighted average noise, dB (mean [SD])	56.05 (4.26)	56.11 (4.33)
Physical activity (%)		
Low	61,195 (18.5)	3,474 (25.4)
Moderate	135,515 (41.1)	5,281 (38.7)
High	133,213 (40.4)	4,908 (35.9)
Healthy diet (%)		
Low	22,733 (6.0)	1,058 (6.5)
Moderate	302,923 (79.9)	12,984 (80.2)
High	53,465 (14.1)	2,155 (13.3)

### Genetic burden of biological functional modules associated with depression

A total of 39 GO terms were enriched by FUMA (*P*_adjusted_ < 0.05) (Supplementary Table S1), such as protein heterodimerization activity (*P*_adjusted_ = 4.23 × 10^−5^), postsynaptic specialization (*P*_adjusted_ = 0.003), glutamatergic synapse (*P*_adjusted_ = 0.006), regulation of immune response (*P*_adjusted_ = 0.034), and MHC class I protein complex (*P*_adjusted_ = 0.043). The PRS of each GO was calculated to estimate the genetic burden of depression corresponding to a specific biological function. The distribution of PRS values is presented in Supplementary Table S2. We additionally assessed the proportion of phenotypic variance explained by individual GO-PRSs, which is provided in Supplementary Table S3.

### Genetic risk profiles of depression

**Figure 2.**
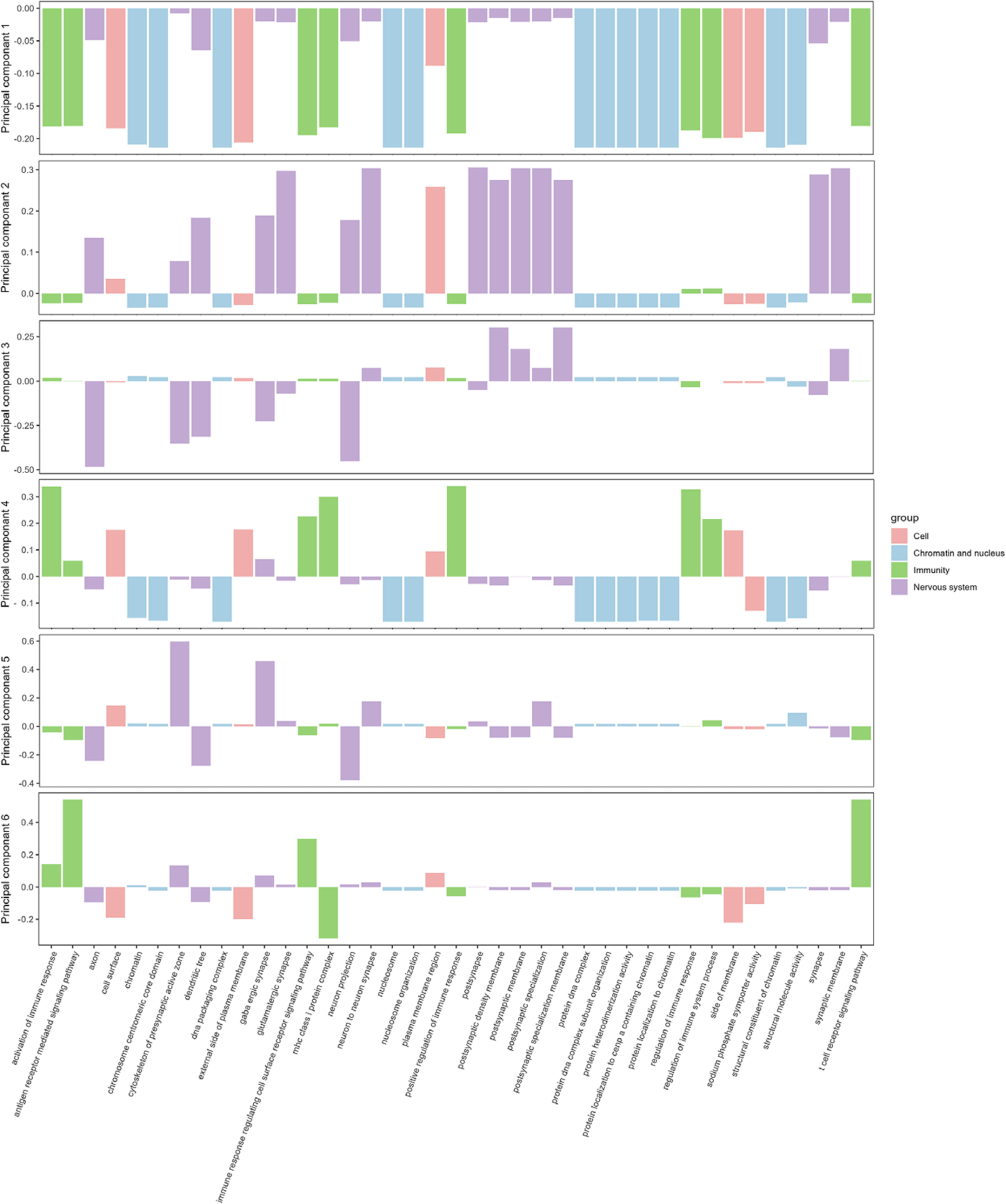
GO-PRS loadings for the first six principal components. Figure 2. long description.

PCA of the 39 GO-PRS revealed that the first six principal components accounted for 91.9% of the total variance (Supplementary Figure S1, Table S4). Principal component 1 explained 52.2% of genetic structure variance, capturing a pattern associated with a decreased genetic risk of depression related to immunity, chromatin and nucleus, and cell. Principal component 2 explained 23.7% of the variance and was characterized by an increased genetic risk of depression related to the nervous system. Principal component 3 explained 5.4% of the variance and was characterized by increased genetic risk of depression related to postsynaptic specialization and a decreased genetic risk related to presynaptic structure and neuronal projection. Principal component 4 explained 4.8% of the variance and captured a pattern associated with an increased genetic risk of depression related to immunity. The loadings of the GO-PRS for the first six principal components are shown in Figure 2.

### Depression genetic pattern of participants

Based on the elbow method, cluster analysis identified three clusters as optimal (Figure S2). The WSS showed a clear inflection point at *k* = 3, indicating that increasing the number of clusters beyond three yielded only marginal improvements in explained variance. The entropy value was 1.05. Accordingly, participants were grouped into three genetic subtypes, each characterized by different biological functions (Supplementary Table S5, Figure S3–S4). Immune-dominant subtype (subtype 1, *N* = 163,417) was characterized by high genetic risk associated with immune and genetic regulation. Neuro-dominant subtype (subtype 2, *N* = 78,393) showed a genetic risk mainly related to neurological functions. Comprehensive-risk subtype (subtype 3, *N =* 168,311) exhibited the highest genetic risk for depression, particularly in neurological, immune, and cellular functions. The Mahalanobis distances between clusters further indicated that the subtypes were distinct: 2.49 (immune-dominant vs. neuro-dominant), 1.61 (immune-dominant vs. comprehensive-risk), and 2.50 (neuro-dominant vs. comprehensive-risk).

### Association between depression genetic risk patterns and the risk of depression and comorbidities

Overall, compared to immune-dominant and neuro-dominant subtypes, individuals in comprehensive-risk subtype exhibited higher prevalence of depression and other diseases, as well as comorbidities (Supplementary Table S6). Compared to immune-dominant and neuro-dominant subtypes, comprehensive-risk subtype was significantly associated with an increased risk of depression (HR: 1.12, 95% CI: 1.08–1.16; HR: 1.10, 95% CI: 1.05–1.15, respectively). We also found significant associations between genetic risk patterns and the risk of diseases following depression (Table 2, Supplementary Table S7). For instance, comprehensive-risk subtype showed higher risks of obesity requiring hospital treatment (HR: 1.16, 95% CI: 1.05–1.28) compared to immune-dominant subtype, and higher risk of ischemic heart diseases (HR: 1.18, 95% CI: 1.00–1.38) compared to neuro-dominant subtype. Moreover, neuro-dominant subtype showed a lower risk of diseases of the digestive system (HR: 0.90, 95% CI: 0.81–0.99) compared to immune-dominant subtype. In contrast, compared to immune-dominant subtype, neuro-dominant subtype was associated with higher risks of anemia (HR: 1.18, 95% CI: 1.02–1.35) following depression.

**Table 2.** Significant associations between depression genetic subtype and depression, and transition from incident depression to subsequent diseases Table 2. long description.

Genetic subtype	Reference subtype	Disease	HR (95% CI)	*P*
Comprehensive-risk	Immune-dominant	Depression	1.12 (1.08–1.16)	2.51 × 10^−11^
Comprehensive-risk	Neuron-dominant	Depression	1.10 (1.05–1.15)	1.09 × 10^−5^
Comprehensive-risk	Immune-dominant	Obesity requiring hospital treatment	1.16 (1.05–1.28)	0.004
Comprehensive-risk	Neuron-dominant	Renal failure	0.83 (0.73–0.95)	0.007
Comprehensive-risk	Immune-dominant	Rheumatoid arthritis and related disorders	1.14 (1.03–1.27)	0.011
Neuron-dominant	Immune-dominant	Anemia	1.18 (1.02–1.35)	0.021
Neuron-dominant	Immune-dominant	Diseases of the digestive system	0.90 (0.81–0.99)	0.031
Neuron-dominant	Immune-dominant	Renal failure	1.15 (1.01–1.32)	0.036
Comprehensive-risk	Immune-dominant	Neurotic disorders	0.93 (0.87–1.00)	0.039
Comprehensive-risk	Neuron-dominant	Ischemic heart diseases	1.18 (1.00–1.38)	0.048

### The impact of genetic pattern on the association between air pollution with depression and comorbidities

The association between PM_2.5_ exposure and the incidence of depression was only detected in individuals with the immune-dominant subtype (Supplementary Table S8). In addition, subtype-specific associations were identified between air pollution exposure and various subsequent diseases after depression (Supplementary Table S9–S11, Figure S5–S7). In immune-dominant subtype, exposure to PM_2.5_ was associated with an increased risk of transitioning from incident depression to ischemic heart disease and diseases of the eye (Table 3). For neuro-dominant subtype, exposure to PM_2.5–10_ increased the risk of transitioning to diabetes, osteoarthritis, and diseases of the eye. For comprehensive-risk subtype, exposure to PM_2.5_ was strongly associated with an elevated risk of transitioning from incident depression to inflammatory bowel disease (IBD), with PM_2.5–10_ also contributing to a higher risk of IBD. In the sensitivity analysis, after excluding road proximity and average noise as covariates, the estimated associations between air pollution and health outcomes became weaker (Supplementary Tables S12–S15).

**Table 3.** Significant associations between air pollution and transition from incident depression to subsequent diseases across various genetic subtypes Table 3. long description.

Air pollutants	Diseases secondary to depression	HR (95% CI)	*P*
Immune-dominant subtype			
PM_2.5_	Ischemic heart diseases	1.28 (1.02–1.60)	0.035
PM_2.5_	Diseases of the eye	1.20 (1.02–1.42)	0.029
PM_2.5_	Osteoarthritis	0.83 (0,72–0.96)	0.012
PM_2.5_	Soft tissue disorders	0.82 (0.68–0.98)	0.030
PM_2.5–10_	Renal failure	1.28 (1.06–1.54)	0.011
Neuro-dominant subtype			
PM_2.5–10_	Diabetes	1.45 (1.01–2.07)	0.042
PM_2.5–10_	Osteoarthritis	1.28 (1.05–1.56)	0.014
PM_2.5–10_	Diseases of the eye	1.37 (1.07–1.74)	0.012
Comprehensive-risk subtype			
PM_2.5_	Inflammatory bowel disease	1.47 (1.07–2.02)	0.019
PM_2.5–10_	Inflammatory bowel disease	1.44 (1.06–1.96)	0.018

### The impact of genetic pattern on the association between life factors with depression and comorbidities

**Figure 3.**
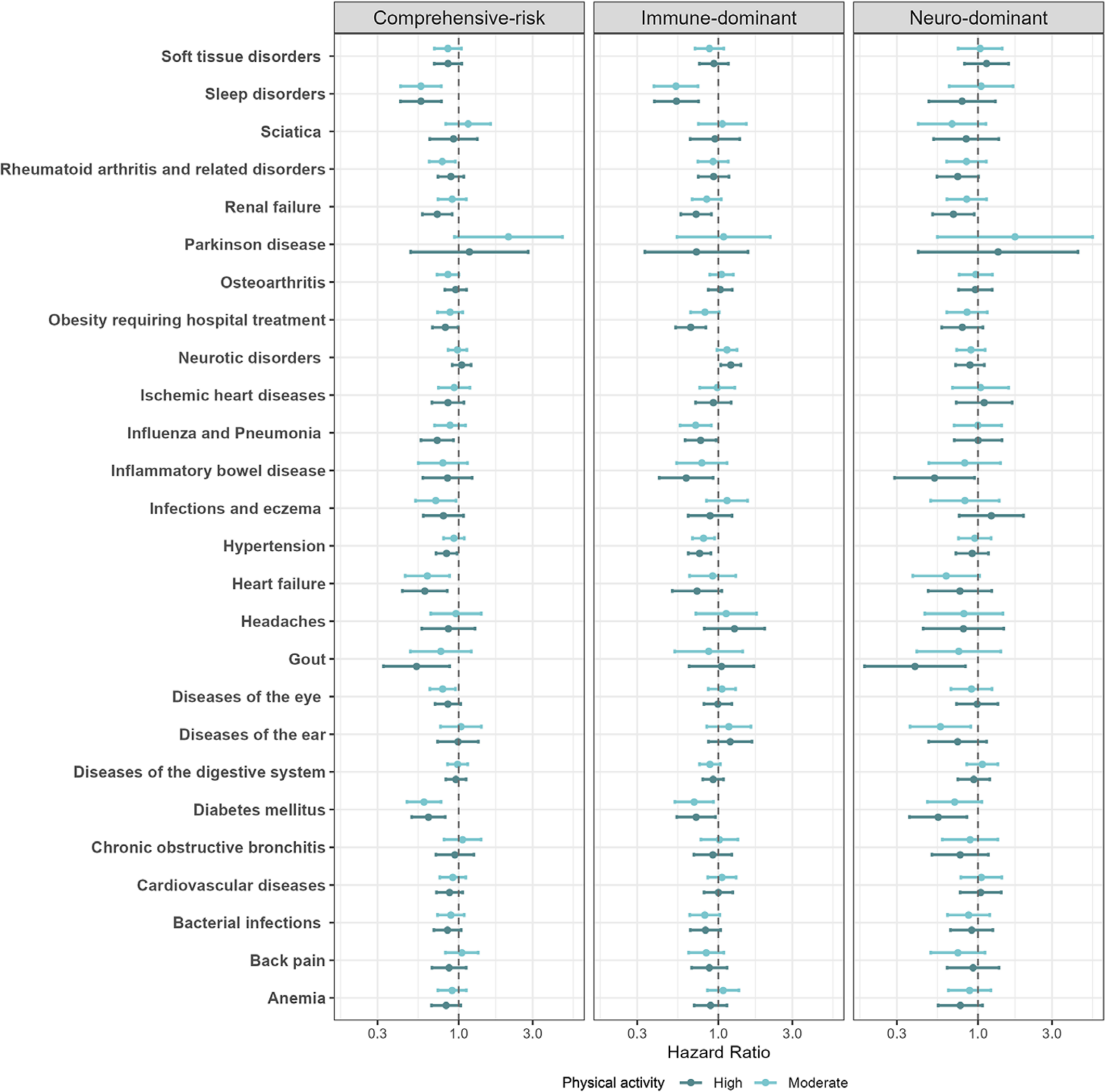
Association between physical activity and secondary diseases of depression across different genetic subtypes. *Note:* *The x-axis represents the hazard ratio (HR), with points and error bars indicating the HR and 95% confidence intervals (CI). The low physical activity was considered the reference. Each panel represents one genetic subtype. HRs are displayed on a logarithmic x-axis for visualization. Figure 3. long description.

A high-level healthy diet was negatively associated with depression across all subtypes, while a moderate-level healthy diet was only associated with depression in comprehensive-risk subtype (Supplementary Table S8). The healthy diet was also negatively associated with the risk of transitioning from depression to ischemic heart diseases in immune-dominant subtype, and headaches in comprehensive-risk subtype (Supplementary Table S16, Figure S8). Physical activity exhibited a protective effect against depression across all clusters (Supplementary Table S8). In immune-dominant and comprehensive-risk subtypes, physical activity was negatively associated with the risk of common chronic diseases such as obesity, diabetes mellitus, hypertension, sleep disorders and renal failure (Supplementary Table S17, Figure 3). In contrast, neuro-dominant subtype showed a more limited response to diet and physical activity, with significant negative associations with physical activity observed for IBD, renal failure, diabetes mellitus and diseases of the eye.

### Differences of correlations between genetic subtypes, environment factors and subsequent diseases after depression

Correlation analyses were performed to assess scale-free associations between genetic subtypes, environmental exposures, and subsequent diseases after depression. The correlations between depression subtypes and diseases ranged from −0.023 to 0.024 (Supplementary Table S18). We also observed subtype-specific differences in correlation patterns across disease outcomes (Supplementary Table S19). For example, the comprehensive-risk subtype exhibited relatively higher correlations with obesity requiring hospital treatment and ischemic heart disease compared with the immune-dominant and neuro-dominant subtypes, respectively. Within each genetic subtype, differences were also observed in the correlations between environmental factors and various subsequent diseases after depression (Supplementary Table S20–S21). For instance, in the immune-dominant subtype, the correlation between a healthy diet and diabetes was lower than its correlation with heart failure, whereas in the comprehensive-risk subtype, the correlation between PM_2.5_ exposure and diabetes was higher than that with heart failure.

## Discussion

This study provides a novel perspective on the genetic complexity of depression by categorizing depression-related genetic loci into distinct biological functional modules. By evaluating the polygenic risk within these modules, we identified unique genetic components that allow us to classify individuals into genetically distinct subtypes. This approach offers novel insights into how genetic subtypes influence depression risk and its associated comorbidities, as well as the association between environmental exposures and disease risk across different subtypes. Overall, this work represents a significant step forward in uncovering the multifaceted nature of depression and its broader health implications.

We employed a GO-based approach to improve biological interpretability. Traditional genome-wide PRS methods yield a single cumulative risk score that aggregates effects across the entire genome, lacking functional specificity. In contrast, our method computed 39 GO-PRSs, each reflecting genetic risk mapped to a distinct biological process. This framework facilitates the dissection of genetic risk across different functional pathways, rather than treating it as a monolithic construct. PCA captured the major axes of genetic variation within the GO-PRS, while clustering analyses identified heterogeneous genetic patterns across individuals, delineating biologically distinct subtypes of depression. Individuals in different subtypes exhibited varying levels of pathway-specific genetic risk. These subtypes capture differences in the overall burden of genetic susceptibility, which may contribute to individual variability in disease onset, progression, and comorbid outcomes.

We identified distinct genetic risk features for depression, which were categorized primarily into immune, neural, and genetic regulatory pathways, revealing the genetic heterogeneity underlying depression. Comprehensive-risk subtype exhibited high genetic risks across all functional categories, representing an accumulation of multiple biological risk factors. Neuro-dominant subtype was marked by elevated neural risks, while immune-dominant subtype showed higher genetic risks mainly related to immune and genetic regulation. The pathogenesis of depression has long been linked to alterations in brain structure, dysfunction in neural circuits, and disruptions in neuroplasticity and neurogenesis (Wolfgang Marx et al., 2023). Its pathophysiology also involves immune and inflammatory processes beyond the central nervous system (Wolfgang Marx et al., 2023). Moreover, genetic regulation further contributes to its pathophysiology (Tena-Campos et al., 2016; Uchida, Yamagata, Seki, & Watanabe, 2018). Our findings suggest that the identified genetic subtypes capture distinct constellations of biological processes implicated in depression, providing a genetic framework that reflects its multisystem pathophysiology.

We observed substantial genetic heterogeneity in depression and its subsequent comorbidities across the identified subtypes. Compared with the immune-dominant and neuro-dominant subtypes, individuals classified into the comprehensive-risk subtype showed a significantly higher incidence of depression and an increased risk of developing a broader spectrum of diseases following depression. For example, the comprehensive-risk subtype exhibited a higher risk of obesity requiring hospital treatment compared with the immune-dominant subtype. This subtype is characterized by concurrently elevated polygenic burden across multiple biological functional domains, including neural-related GO categories. Previous studies have reported associations between neural-related genetic variation and vulnerability to psychiatric and metabolic conditions (Milaneschi, Simmons, van Rossum, & Penninx, 2019), which supports our findings. In addition, the comprehensive-risk subtype demonstrated a higher risk of transitioning from depression to ischemic heart disease compared with the neuro-dominant subtype. Given that this subtype has higher immune-related GO-PRS, this association may reflect shared genetic architecture involving immune and inflammatory pathways. Such an interpretation aligns with existing evidence implicating immune-related genetic susceptibility in both depression and ischemic heart disease (Beurel, Toups, & Nemeroff, 2020; Wu, Ying, et al., 2024). Importantly, this enrichment-based interpretation does not imply direct causality, but rather highlights overlapping genetic risk profiles across conditions.

Individuals within the immune-dominant subtype showed a higher risk of transitioning from incident depression to digestive system diseases compared with the neuro-dominant subtype. Prior literature has reported strong links between immune and inflammatory pathways and both depression and digestive system disorders (Wu, Ou, et al., 2024; Xie et al., 2022). In this context, our findings suggest that the immune-dominant subtype captures a pattern of shared genetic susceptibility enriched for immune-related biological functions, which may underlie the observed comorbidity patterns. However, we cannot exclude alternative explanations, including reverse causation or the influence of unmeasured confounding factors. Future studies incorporating longitudinal biomarker data will be necessary to clarify temporal and causal relationships. Finally, the neuro-dominant subtype exhibited a higher risk of anemia following depression compared with the immune-dominant subtype. Previous studies have reported associations between depression and lower levels of hemoglobin, ferritin, folate, and vitamin B12, particularly in older populations (Li et al., 2024). Our findings indicate that genetic susceptibility enriched for neural-related biological functions may co-occur with an increased risk of anemia after depression, potentially reflecting shared genetic or behavioral risk profiles. However, because nutritional status, metabolic factors, and related intermediate phenotypes were not directly assessed, the present findings primarily reflect genetic co-enrichment across neural-related biological functions, and thus further studies are needed to elucidate the underlying biological mechanisms.

Previous studies have suggested that air pollutants can stimulate pro-inflammatory immune responses in various immune cells, leading to disrupted immune tolerance and impaired antibacterial and antiviral immunity (Glencross et al., 2020). Moreover, experimental research has reported that air pollution exposure can induce neurotoxic or neuroinflammatory responses (Pastor-Belda et al., 2019; Peiffer et al., 2013). Therefore, individuals with elevated genetic risks in immune- or neuron-related pathways may be more susceptible to air pollution, consistent with prior evidence implicating these biological processes. The association between air pollution exposure and ischemic heart disease following depression was observed specifically in individuals with the immune-dominant genetic subtype. Given that immune-related genetic susceptibility has been implicated in both depression and ischemic heart disease (Beurel, Toups, & Nemeroff, 2020; Wu, Ying, et al., 2024), it is plausible that associations between air pollution and disease risk are more readily observed in individuals with this genetic background. For neuro-dominant subtype, exposure to PM_2.5–10_ was linked to an increased risk of diabetes mellitus following depression. Prior literature has reported that certain neural development-related loci may exert pleiotropic influences on both depression and type 2 diabetes (Baranova et al., 2025; Maina et al., 2023). For comprehensive-risk subtype, air pollution exposure was strongly associated with increased risks of IBD. The shared loci linked to depression and IBD implicate genes regulating immune function and neural plasticity and development (Frenkel et al., 2020; Zhou et al., 2025). The accumulation of genetic risk across immune, neurological, and regulatory pathways in this subtype may partially explain why the association between air pollution and IBD was more evident in this group.

Physical activity and diet exhibited associations with a lower risk of depression across all subtypes. In the immune-dominant and comprehensive-risk subtypes, these lifestyle factors were also associated with a lower risk of transition from depression to a broader range of subsequent diseases, including metabolic, immune, respiratory, and cardiovascular diseases. In contrast, among individuals in the neuro-dominant subtype, inverse associations were mainly observed for specific conditions such as diabetes, IBD, and renal failure. This pattern may reflect a relatively weaker modulation by lifestyle factors in this subtype due to its underlying genetic architecture. Regular exercise training exerts a systemic anti-inflammatory effect through multiple pathways, such as improved regulation of inflammatory signaling, reduction of dysfunctional adipose tissue, enhanced oxygenation, strengthened innate immune function, and better balance of oxidized lipids (Nieman & Wentz, 2019). A healthy diet is associated with reduced levels of inflammatory mediators such as C-reactive protein (CRP) and TNF-α (Di Giosia et al., 2022). Additionally, dietary patterns can modulate the gut microbiome, which in turn influences host health by regulating immune function, including the recruitment and differentiation of immune cells (Di Giosia et al., 2022).

Previous studies have attempted to identify genetic heterogeneity based on disease-associated single nucleotide polymorphisms and comorbidity patterns (Gezsi et al., 2024; Randall et al., 2009). However, these approaches remain limited in assessing the risk of diseases and their secondary symptoms and in providing a comprehensive understanding of pathogenesis. In the present study, we employed PCA to capture the major genetic components of depression from a broad set of GO-PRS and utilized cluster analysis to classify the samples into various genetic subtypes. This pathway-based approach provides biologically informed insights into how distinct genetic modules may relate to depression and its comorbidities. Unlike traditional genome-wide PRS, which yields a single aggregate risk score and lacks functional context, our method facilitates biologically interpretable stratification by mapping genetic risk to various functional pathways. Moreover, our findings suggest that genetic subtypes may differ in their associations with environmental factors, highlighting the potential role of genetic susceptibility in shaping individual responses to lifestyle-related exposures.

Our findings offer several important clinical implications. First, individuals with different genetic subtypes may benefit from tailored therapeutic approaches. For instance, individuals in immune-dominant subtype, characterized by heightened immune-related genetic risk and greater vulnerability to air pollution, may benefit from immune-modulating therapies alongside environmental risk mitigation. Comprehensive-risk subtype, with elevated risks across multiple biological domains, may necessitate combination approaches targeting both immune and neurological pathways. Second, the varying effectiveness of lifestyle interventions across subtypes suggests the need for personalized environmental strategies. Notably, individuals in immune-dominant and comprehensive-risk subtypes experienced stronger protective effects from healthy lifestyle factors, highlighting these modifiable behaviors as potential intervention points in specific genetic contexts. Stratifying individuals according to genetically informed subtypes may provide a framework for understanding differential vulnerability pathways and for refining clinical risk assessment. These subtypes, reflecting distinct constellations of biological processes, may help identify individuals who are more susceptible to environmental stressors or who exhibit characteristic trajectories of comorbidity progression. Such information can support the development of more targeted monitoring strategies and earlier preventive interventions, and may also inform adjunctive strategies tailored to the specific biological risk profiles of each subtype, ultimately contributing to improved long-term prognosis for depression and its associated chronic conditions.

This study has some limitations. First, the UK Biobank cohort is not representative of the general population in terms of sociodemographic, physical, lifestyle, and health-related characteristics (Fry et al., 2017). However, its large size and heterogeneity of exposure measures provide valid scientific inferences of associations between exposures and health conditions that are generalizable to other populations. Second, the participants in our research were mainly white British, so our findings should be used with caution when applying to individuals from other races and regions. Additionally, PRS typically explain only a small proportion of the total heritability. They are also subject to population-specific biases and do not account for environmental factors or gene–environment interactions (Schwarzerova et al., 2024). Furthermore, the GWAS summary statistics used to construct the PRS partially overlap with the target sample. LD Score Regression indicated minimal inflation due to this non-independence (intercept = 1.0011; ratio = 0.0019). Nonetheless, future studies using fully independent GWAS datasets would further reduce potential bias. Moreover, this study lacks independent replication, which may affect the generalizability of the findings. Future research is needed to validate these results in new, independent datasets. For subtypes that exhibit elevated genetic risks across multiple biological domains, it remains unclear whether these genetic risk factors act independently or synergistically, and further research is needed to elucidate their potential interactions. The lack of statistical significance may be partly due to the limited sample size within each subtype. Future studies with larger sample sizes and independent replication cohorts are warranted to validate these findings.

In conclusion, we systematically explored the genetic pattern of depression using a clustering approach based on the genetic risk of biological functional modules. By identifying distinct genetic components and classifying individuals into various subtypes with unique genetic characteristics, our findings highlight the genetic heterogeneity of depression and its comorbidities. We also underscore how genetic components influence individual responses to environmental factors. This approach not only deepens our understanding of the pathogenesis of depression and its comorbidities but also provides insights into developing personalized prevention and treatment strategies.

## Supporting information

10.1017/S0033291726104140.sm001 Pan et al. supplementary material 1 Pan et al. supplementary material

10.1017/S0033291726104140.sm002 Pan et al. supplementary material 2 Pan et al. supplementary material

## Data Availability

The UK Biobank data are available through the UK Biobank Access Management System https://www.ukbiobank.ac.uk/. The code supporting the main analysis is publicly available at the following URL: https://github.com/ChuyuPan/Code-for-PSM-D-25-01198/tree/main.

## Supplementary material

The supplementary material for this article can be found at http://doi.org/10.1017/S0033291726104140.

## Long descriptions

### Figure 1. Long description

Starting from the top, the flowchart begins with ‘Genetic loci with P less than 5 times 10 to the minus 8 in genome-wide meta-analysis of depression (N equals 807,553)’. Downward arrow leads to ‘Functional enrichment analysis: Gene Ontology G O associated with depression’, then to ‘Calculation of G O-based Polygenic risk score P R S’. Next is ‘Principal component analysis P C A: genetic component of depression’, followed by ‘Cluster analysis: three genetic subtypes of depression’. From this box, a leftward arrow leads to ‘Chi-square test: Prevalence of depression and 26 diseases across genetic subtypes’. To the right, two boxes branch off: ‘UK Biobank participants with genetic data (N equals 487,409)’ and ‘UK Biobank participants free of depression at baseline with data on environmental factors (air pollution, diet, physical activity) and covariates (N equals 410,121)’. The central flow continues to ‘Unidirectional multi-state Markov proportional hazards model’, which branches into two final boxes: ‘Associations between genetic risk pattern and the risk of incident depression and subsequent health conditions’ and ‘Associations between environmental factors and the risk of incident depression and subsequent health conditions across genetic subtypes’.

Navigate back to Figure 1..

### Table 1. Long description

From the top row, columns are: variable, subcategory, Control group count and percent, Depression group count and percent. Sex, male: Control 189,344 (48.2 percent), Depression 6,372 (37.3 percent). Age 65 years or older: Control 69,549 (17.7 percent), Depression 3,041 (17.8 percent). Age under 65: Control 323,500 (82.3 percent), Depression 14,031 (82.2 percent). T D I mean and standard deviation: Control minus 1.40 (3.03), Depression minus 0.63 (3.32). Education, university or college degree: Control 141,138 (35.9 percent), Depression 4,528 (26.5 percent). Other education: Control 251,911 (64.1 percent), Depression 12,544 (73.5 percent). Household income low: Control 185,297 (47.1 percent), Depression 11,114 (65.1 percent). Moderate: Control 103,789 (26.4 percent), Depression 3,619 (21.2 percent). High: Control 103,963 (26.5 percent), Depression 2,339 (13.7 percent). Ever drank alcohol: Control 378,256 (96.2 percent), Depression 16,256 (95.2 percent). Ever smoked: Control 177,229 (45.1 percent), Depression 9,336 (54.7 percent). Cluster 1: Control 156,928 (39.9 percent), Depression 6,489 (38.0 percent). Cluster 2: Control 75,209 (19.1 percent), Depression 3,184 (18.7 percent). Cluster 3: Control 160,912 (40.9 percent), Depression 7,399 (43.3 percent). N O sub 2 high: Control 189,381 (48.9 percent), Depression 8,939 (53.0 percent). P M sub 2.5 high: Control 176,816 (49.3 percent), Depression 9,153 (54.9 percent). P M sub 2.5–10 high: Control 177,763 (49.5 percent), Depression 8,349 (50.1 percent). Distance to major roads, 1 per kilometer mean and standard deviation: Control 0.01 (0.02), Depression 0.01 (0.01). 24-hour weighted average noise, d B mean and standard deviation: Control 56.05 (4.26), Depression 56.11 (4.33). Physical activity low: Control 61,195 (18.5 percent), Depression 3,474 (25.4 percent). Moderate: Control 135,515 (41.1 percent), Depression 5,281 (38.7 percent). High: Control 133,213 (40.4 percent), Depression 4,908 (35.9 percent). Healthy diet low: Control 22,733 (6.0 percent), Depression 1,058 (6.5 percent). Moderate: Control 302,923 (79.9 percent), Depression 12,984 (80.2 percent). High: Control 53,465 (14.1 percent), Depression 2,155 (13.3 percent).

Navigate back to Table 1..

### Figure 2. Long description

From top to bottom, each panel displays loadings for principal components one through six. The x-axis lists biological terms, including ‘antigen processing and presentation’, ‘cell cycle’, ‘chromatin organization’, ‘immune response’, and ‘synapse organization’. Bars are color-coded: blue for cell, pink for chromatin and nucleus, green for immunity, and purple for nervous system. Principal component one shows strong negative loadings for cell-related terms and positive for nervous system. Principal component two has prominent positive loadings for nervous system and negative for cell and immunity. Principal component three features mixed loadings, with nervous system dominant. Principal component four highlights immunity and cell groups. Principal component five shows moderate positive loadings for nervous system and cell, with negative for immunity. Principal component six displays strong positive loadings for immunity and cell, with negative for chromatin and nucleus. The legend at the right clarifies group colors.

Navigate back to Figure 2..

### Table 2. Long description

The table contains ten rows, each representing a comparison between genetic subtypes and disease outcomes. Columns are, from left to right: genetic subtype, reference subtype, disease, hazard ratio with 95 percent confidence interval, and p-value. Row 1: Comprehensive-risk vs Immune-dominant for depression, hazard ratio 1.12 (1.08 to 1.16), p-value 2.51 times 10 to the minus 11. Row 2: Comprehensive-risk vs Neuron-dominant for depression, hazard ratio 1.10 (1.05 to 1.15), p-value 1.09 times 10 to the minus 5. Row 3: Comprehensive-risk vs Immune-dominant for obesity requiring hospital treatment, hazard ratio 1.16 (1.05 to 1.28), p-value 0.004. Row 4: Comprehensive-risk vs Neuron-dominant for renal failure, hazard ratio 0.83 (0.73 to 0.95), p-value 0.007. Row 5: Comprehensive-risk vs Immune-dominant for rheumatoid arthritis and related disorders, hazard ratio 1.14 (1.03 to 1.27), p-value 0.011. Row 6: Neuron-dominant vs Immune-dominant for anemia, hazard ratio 1.18 (1.02 to 1.35), p-value 0.021. Row 7: Neuron-dominant vs Immune-dominant for diseases of the digestive system, hazard ratio 0.90 (0.81 to 0.99), p-value 0.031. Row 8: Neuron-dominant vs Immune-dominant for renal failure, hazard ratio 1.15 (1.01 to 1.32), p-value 0.036. Row 9: Comprehensive-risk vs Immune-dominant for neurotic disorders, hazard ratio 0.93 (0.87 to 1.00), p-value 0.039. Row 10: Comprehensive-risk vs Neuron-dominant for ischemic heart diseases, hazard ratio 1.18 (1.00 to 1.38), p-value 0.048.

Navigate back to Table 2..

### Table 3. Long description

Starting from the top, the table is divided into three genetic subtypes: Immune-dominant, Neuro-dominant, and Comprehensive-risk. For the Immune-dominant subtype, P M sub 2.5 is associated with ischemic heart diseases (hazard ratio 1.28, 95 percent confidence interval 1.02 to 1.60, p-value 0.035), diseases of the eye (1.20, 1.02 to 1.42, 0.029), osteoarthritis (0.83, 0.72 to 0.96, 0.012), and soft tissue disorders (0.82, 0.68 to 0.98, 0.030). P M sub 2.5–10 is linked to renal failure (1.28, 1.06 to 1.54, 0.011). For the Neuro-dominant subtype, P M sub 2.5–10 is associated with diabetes (1.45, 1.01 to 2.07, 0.042), osteoarthritis (1.28, 1.05 to 1.56, 0.014), and diseases of the eye (1.37, 1.07 to 1.74, 0.012). For the Comprehensive-risk subtype, P M sub 2.5 is linked to inflammatory bowel disease (1.47, 1.07 to 2.02, 0.019), and P M sub 2.5–10 is also linked to inflammatory bowel disease (1.44, 1.06 to 1.96, 0.018). Each row presents the air pollutant, the secondary disease, the hazard ratio with its 95 percent confidence interval, and the p-value.

Navigate back to Table 3..

### Figure 3. Long description

From left to right, the panels are labeled Comprehensive-risk, Immune-dominant, and Neuro-dominant. Each panel lists 25 diseases vertically, starting with Soft tissue disorders at the top and ending with Anemia at the bottom. For each disease, two horizontal lines represent hazard ratios for high and moderate physical activity, with points and error bars indicating the hazard ratio and 95 percent confidence interval. The x-axis is labeled Hazard Ratio and ranges from 0.3 to 3.0, displayed logarithmically. The reference group is low physical activity. Most hazard ratios for high and moderate activity are below 1.0, especially for diseases like sleep disorders, ischemic heart diseases, and diabetes mellitus, indicating reduced risk. Some diseases, such as infections and eczema, show hazard ratios near or above 1.0. The legend at the bottom identifies line colors for high and moderate physical activity.

Navigate back to Figure 3..
