# Supplementary material for: Genetic heterogeneity affects the risk of incident depression, comorbidity, and response to environment: A prospective trajectory study
Source: Psychol Med. 2026 Jun 22;56:e205. doi: 10.1017/S0033291726104140 (PMC13319487; doi:10.1017/S0033291726104140)
Supplement: Pan et al. supplementary material 1 — Pan et al. supplementary material [file S0033291726104140sup001.docx]

**Supplementary methods**

**Definition of diseases in the UK Biobank**

| Disease | ICD-10 codes |
| --- | --- |
| Depression | F32-F33 |
| Obesity requiring hospital treatment | E66 |
| Diabetes | E10-E14 |
| Diseases of the digestive system | K00–K93 |
| Sleep disorders | G47 |
| Headaches | G43-G44 |
| Parkinson disease | G20 |
| Back pain | M54 |
| Rheumatoid arthritis and related disorders | M05-M06, M08, M13, M30-M35, M45 |
| Osteoarthritis | M15–M19 |
| Chronic obstructive bronchitis | J43-J44, J47 |
| Heart failure | I50 |
| Anemia | D50-D64 |
| Ischemic heart diseases | I20-I25 |
| Hypertension | I10-I15 |
| Inflammatory bowel disease | K50-K52 |
| All cardiovascular disease | I20-I21, I25, I48, I50, I60, I61, I63, I64 |
| Bacterial infections | A01-A79 |
| Neurotic disorders | F40-F48 |
| Diseases of the eye | H00-H59 |
| Diseases of the ear | H60-H99 |
| Influenza and Pneumonia | J09-J18 |
| Infections and eczema | L00-L08, L20-L30 |
| Gout | M10 |
| Sciatica | M50-M51 |
| Soft tissue disorders | M60-M79 |
| Renal failure | N17-N19 |

**Definition of environmental factor**

***Air pollution***

The air pollution data were derived from the European Study of Cohorts for Air Pollution Effects (ESCAPE) project, which covered 20 European regions for particulate matter (PM) research and 32 European regions for nitrogen dioxide (NO_2_) and nitrogen oxides (NO_x_) research(Beelen et al., 2013; Eeftens et al., 2012). Land-use regression (LUR) models were used to assess individual participants’ residential air pollution exposure based on their home addresses(Beelen et al., 2013; Eeftens et al., 2012). Specific in this study, NO_2_, PM_2.5_ (particulate matter with aerodynamic diameter ≤ 2.5μm), and PM_2.5-10_ (2.5μm< particulate matter with aerodynamic diameter ≤ 10μm) were used as primary air pollution indicators. The exposure levels for NO_2,_ PM_2.5_ and PM_2.5-10_ were determined using the 2010 annual average concentrations.

***Diet***

Diet was assessed using a healthy diet score based on the Mediterranean diet and heart-healthy dietary recommendations for reducing the risk of chronic diseases(Lourida et al., 2019; Mozaffarian, 2016). This score included seven components: fruits, vegetables, fish, processed meat, unprocessed red meat, whole grains, and refined grains. The healthy diet score was calculated by summing the scores for each of the seven food components consumed by each participant, with a range from 0 to 7 (Lourida et al., 2019). Higher scores indicated healthier dietary patterns. The healthy diet scores were classified into three categories: low (0-1 points), moderate (2-5 points), and high (6-7 points) diet scores. According to previous studies, this classification method could accurately reflect the characteristics of different dietary quality groups, enabling a comprehensive exploration of the relationship between dietary patterns and various chronic diseases (Schulz, Weinhold, Schmid, Nöthen, & Nöthlings, 2023).

***Physical activity***

Physical activity was evaluated using International Physical Activity Questionnaire (IPAQ), and participants were classified into three activity levels: high, moderate and low (Committee, 2005). The high IPAQ group was defined as engaging in at least one additional hour of moderat×10-intensity activity above basal levels daily, or at least half an hour of vigorous-intensity activity above basal levels daily. The moderate group was defined as doing some activity, roughly equivalent to 30 minutes of moderate-intensity physical activity on most days. The low IPAQ group included individuals who did not meet the criteria for either the high or moderate activity groups.

**Covariates**

The age was categorized into <65 years and ≥65 years (Tahira, Verjovski-Almeida, & Ferreira, 2021; Yang, Wang, Huang, Kelly, & Li, 2023). Education attainment was synthesized into “university or college degree” and “others”(Cheng et al., 2024; Gao, Jiang, Huang, Guo, & Huang, 2023). The socioeconomic status was evaluated according to household income and the social deprivation level, respectively. Participants with annual household income below £18 000 were classified as having low income, those with income between £18 000 and £51 999 were categorized as having moderate income, and individuals with income exceeding £51 999 were considered to have high income(Gao et al., 2023; Schorr et al., 2025). Social deprivation level was defined based on the Townsend Deprivation Index (TDI). The 24-h weighted average noise was calculated with a 5 and 10 decibel (dB) penalty added to evening and night time, respectively. Proximity to major road was indicated by the inverse distance to the nearest major road. Additionally, diet and exercise were considered in the sensitivity analysis.

**Reference**

Beelen, R., Hoek, G., Vienneau, D., Eeftens, M., Dimakopoulou, K., Pedeli, X., . . . Hoogh, K. d. (2013). Development of NO2 and NOx land use regression models for estimating air pollution exposure in 36 study areas in Europe – The ESCAPE project. *Atmospheric Environment, 72*, 10-23. doi:10.1016/j.atmosenv.2013.02.037

Cheng, B., Pan, C., Cai, Q., Liu, L., Cheng, S., Yang, X., . . . Zhang, F. (2024). Long-term ambient air pollution and the risk of musculoskeletal diseases: A prospective cohort study. *J Hazard Mater, 466*, 133658. doi:10.1016/j.jhazmat.2024.133658

Committee, I. R. (2005). Guidelines for data processing and analysis of the International Physical Activity Questionnaire (IPAQ)-short and long forms. [*http://www*](http://www)*. ipaq. ki. se/scoring. pdf*.

Eeftens, M., Beelen, R., de Hoogh, K., Bellander, T., Cesaroni, G., Cirach, M., . . . Hoek, G. (2012). Development of Land Use Regression models for PM(2.5), PM(2.5) absorbance, PM(10) and PM(coarse) in 20 European study areas; results of the ESCAPE project. *Environ Sci Technol, 46*(20), 11195-11205. doi:10.1021/es301948k

Gao, X., Jiang, M., Huang, N., Guo, X., & Huang, T. (2023). Long-Term Air Pollution, Genetic Susceptibility, and the Risk of Depression and Anxiety: A Prospective Study in the UK Biobank Cohort. *Environ Health Perspect, 131*(1), 17002. doi:10.1289/ehp10391

Lourida, I., Hannon, E., Littlejohns, T. J., Langa, K. M., Hyppönen, E., Kuzma, E., & Llewellyn, D. J. (2019). Association of Lifestyle and Genetic Risk With Incidence of Dementia. *Jama, 322*(5), 430-437. doi:10.1001/jama.2019.9879

Mozaffarian, D. (2016). Dietary and Policy Priorities for Cardiovascular Disease, Diabetes, and Obesity: A Comprehensive Review. *Circulation, 133*(2), 187-225. doi:10.1161/circulationaha.115.018585

Schorr, K., Rodriguez-Girondo, M., den Berg, N. V., de Groot, L. C., Slagboom, P. E., & Beekman, M. (2025). Unhealthful plant-based diet associates with frailty risk predominantly in men with low income from the UK Biobank cohort. *J Nutr Health Aging, 29*(3), 100463. doi:10.1016/j.jnha.2024.100463

Schulz, C. A., Weinhold, L., Schmid, M., Nöthen, M. M., & Nöthlings, U. (2023). Analysis of associations between dietary patterns, genetic disposition, and cognitive function in data from UK Biobank. *Eur J Nutr, 62*(1), 511-521. doi:10.1007/s00394-022-02976-y

Tahira, A. C., Verjovski-Almeida, S., & Ferreira, S. T. (2021). Dementia is an age-independent risk factor for severity and death in COVID-19 inpatients. *Alzheimers Dement, 17*(11), 1818-1831. doi:10.1002/alz.12352

Yang, T., Wang, J., Huang, J., Kelly, F. J., & Li, G. (2023). Long-term Exposure to Multiple Ambient Air Pollutants and Association With Incident Depression and Anxiety. *JAMA Psychiatry, 80*(4), 305-313. doi:10.1001/jamapsychiatry.2022.4812

**Supplementary Figure**


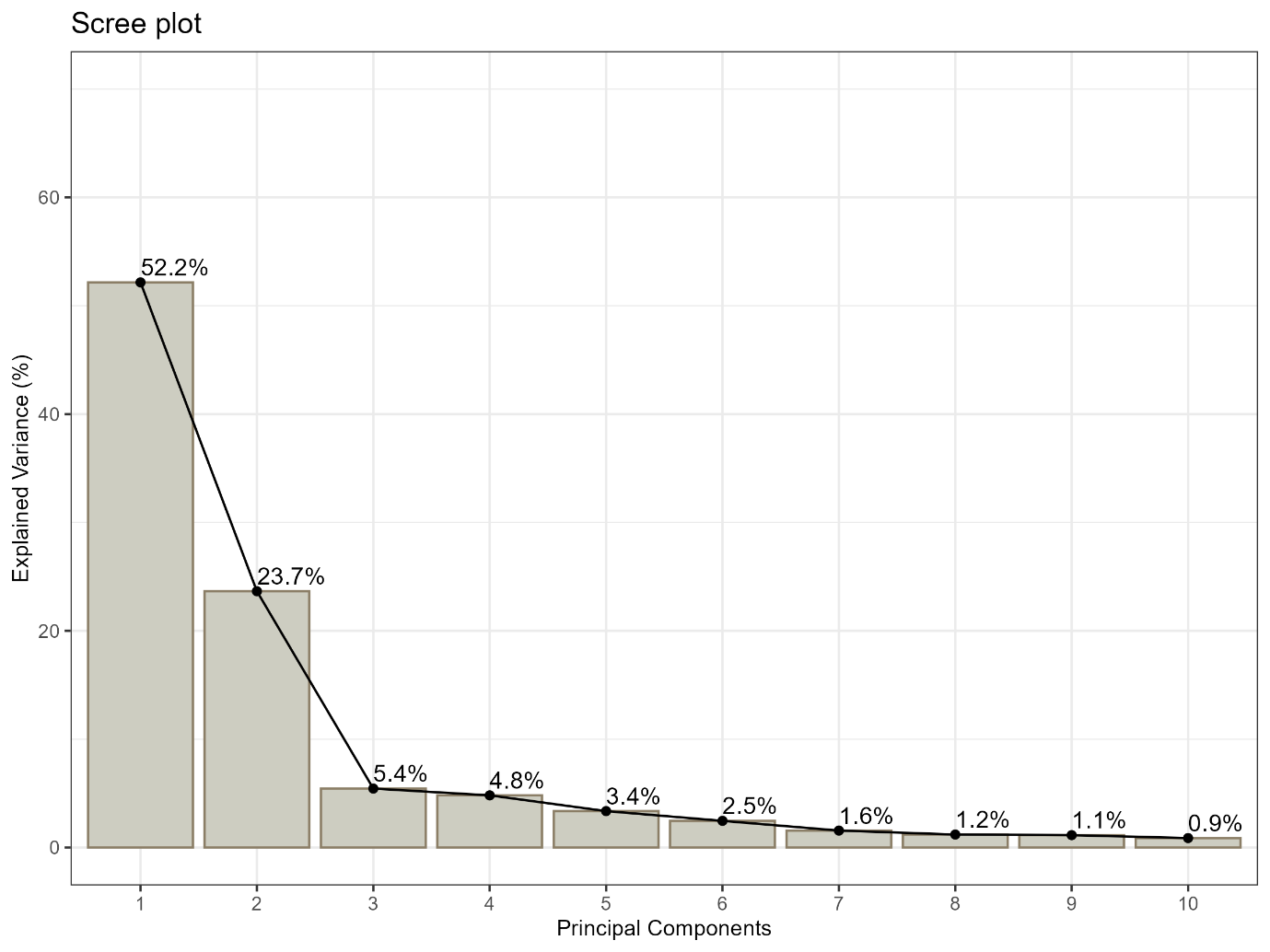


**Figure S1.** Scree plot of principal component analysis.


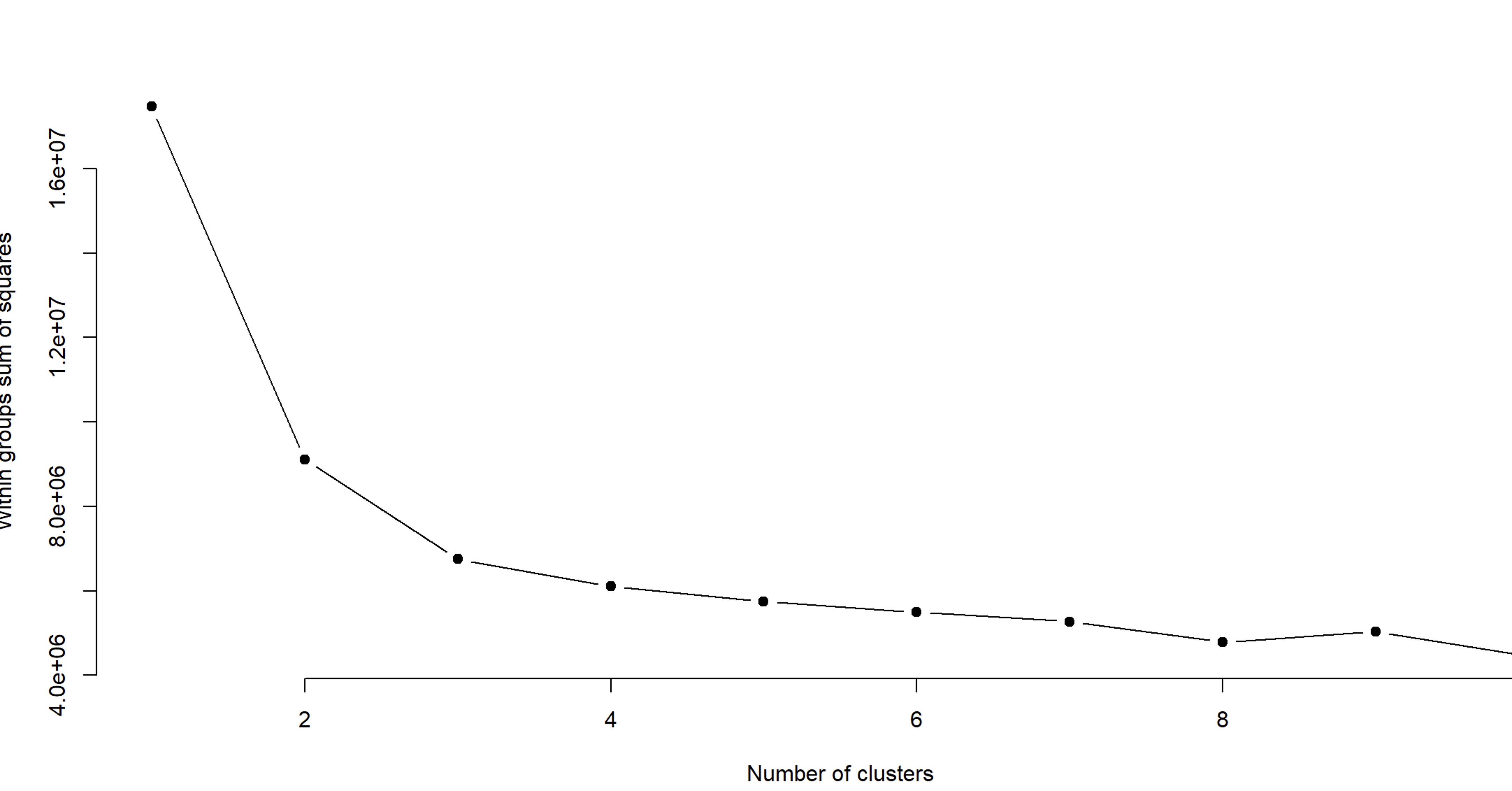


**Figure S2.** Results of elbow methods.


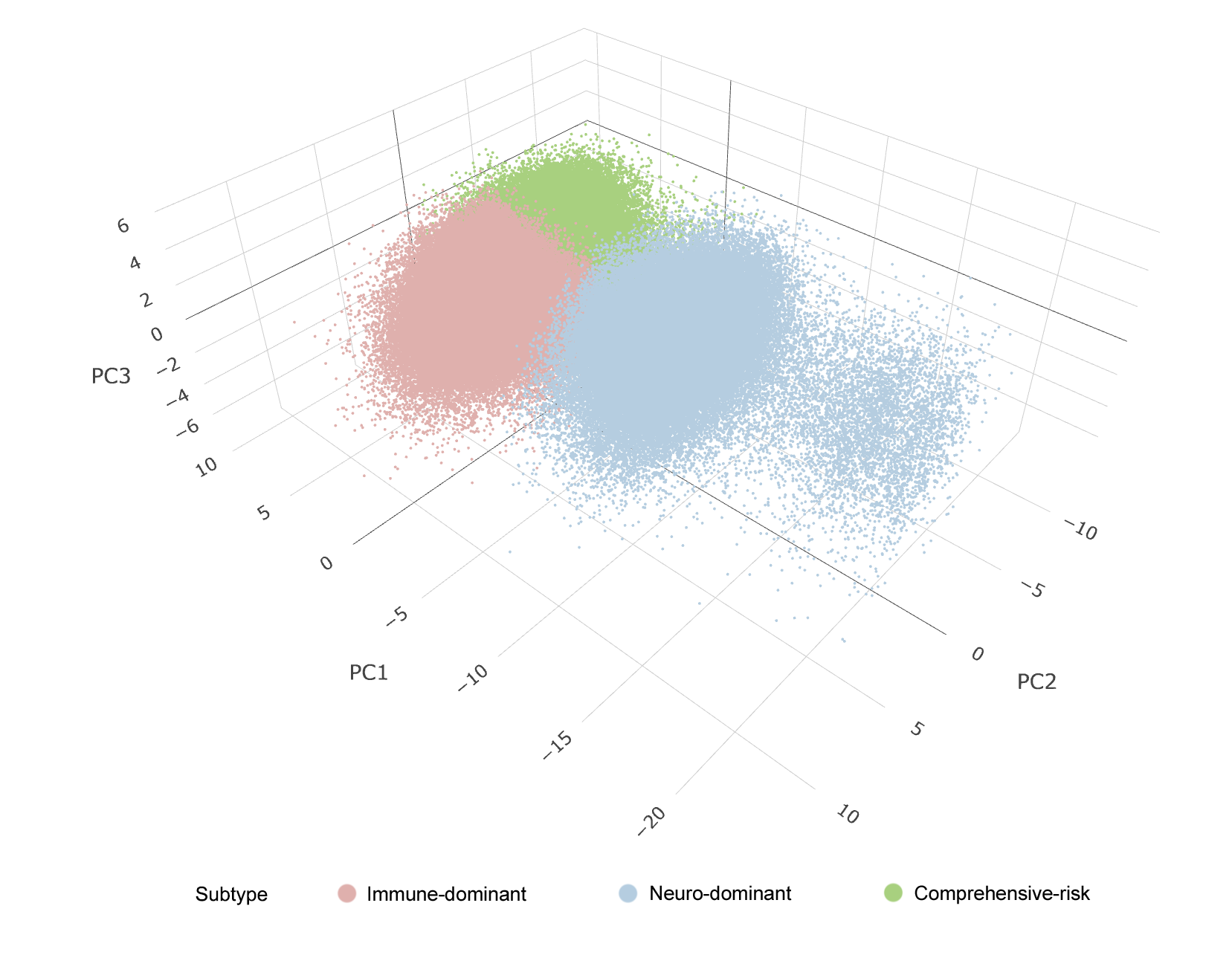


**Figure S3**. Scatter plot of cluster analysis.


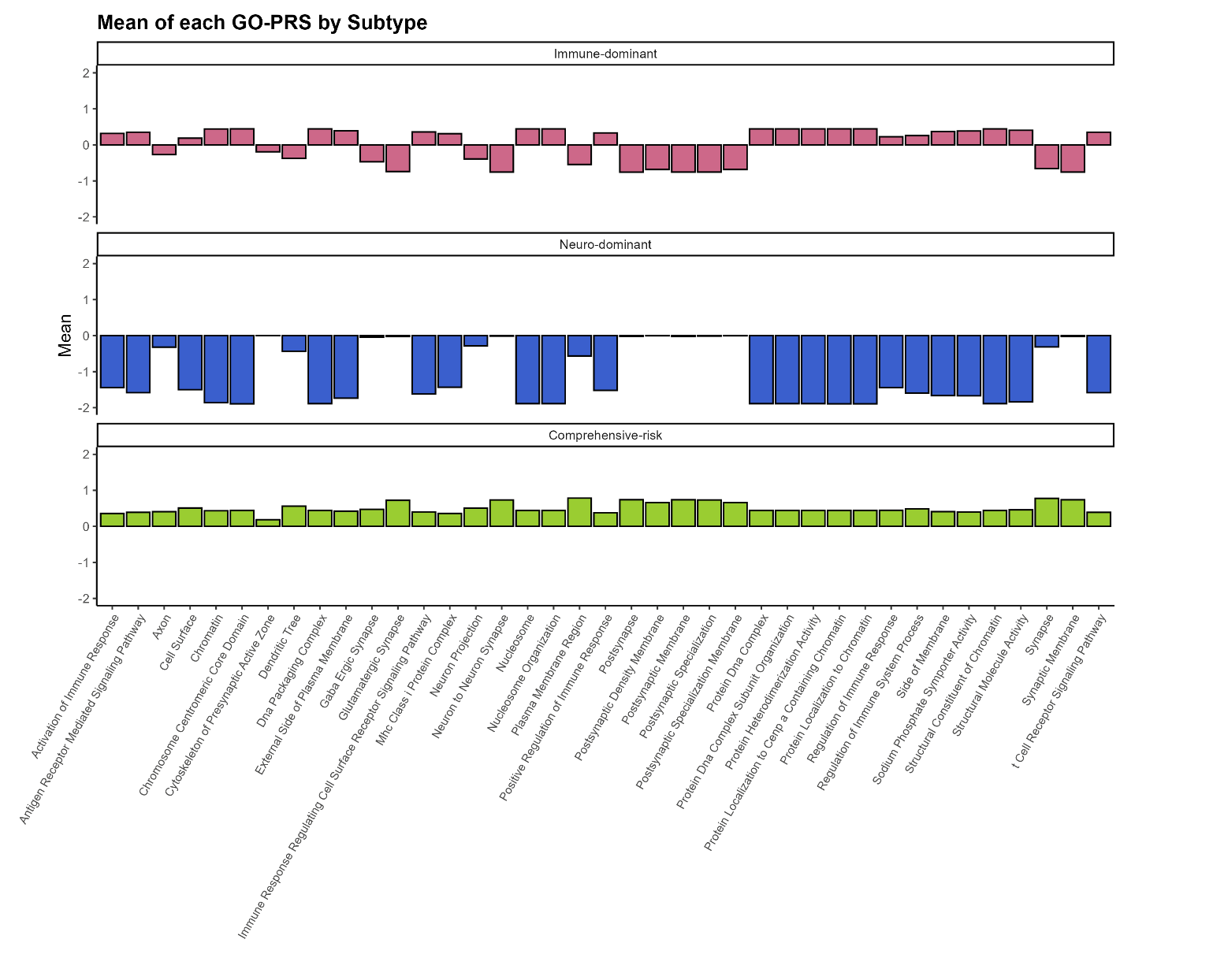


**Figure S4.** Distribution of standardized GO-PRS means across genetic subtypes.

**
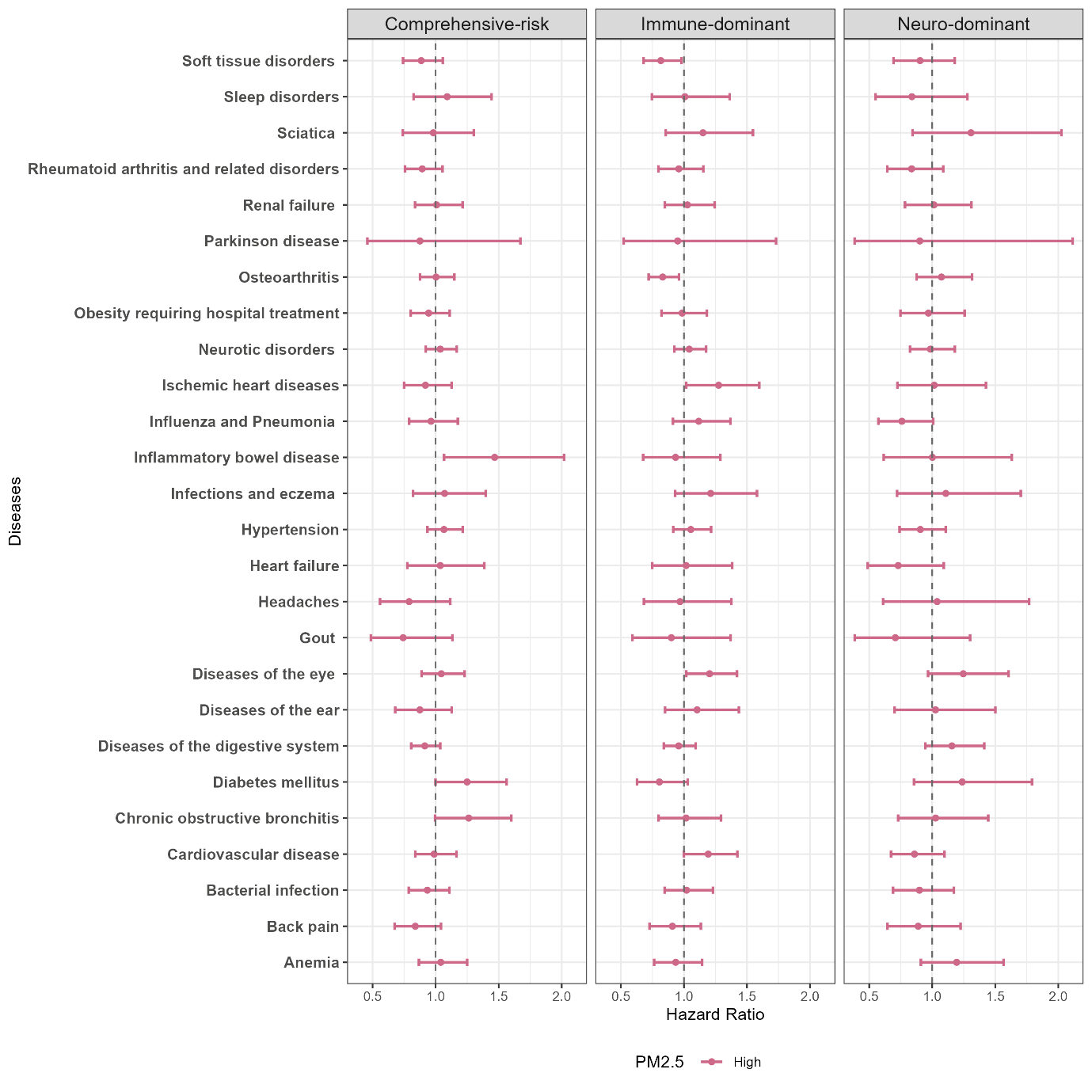
**

**Figure S5**. Association between PM_2.5_ exposure and secondary diseases of depression across different genetic subtypes.

***** The x-axis represents the hazard ratio (HR), with points and error bars indicating the HR and 95% confidence intervals (CI). The low PM_2.5_ exposure was considered reference.

**
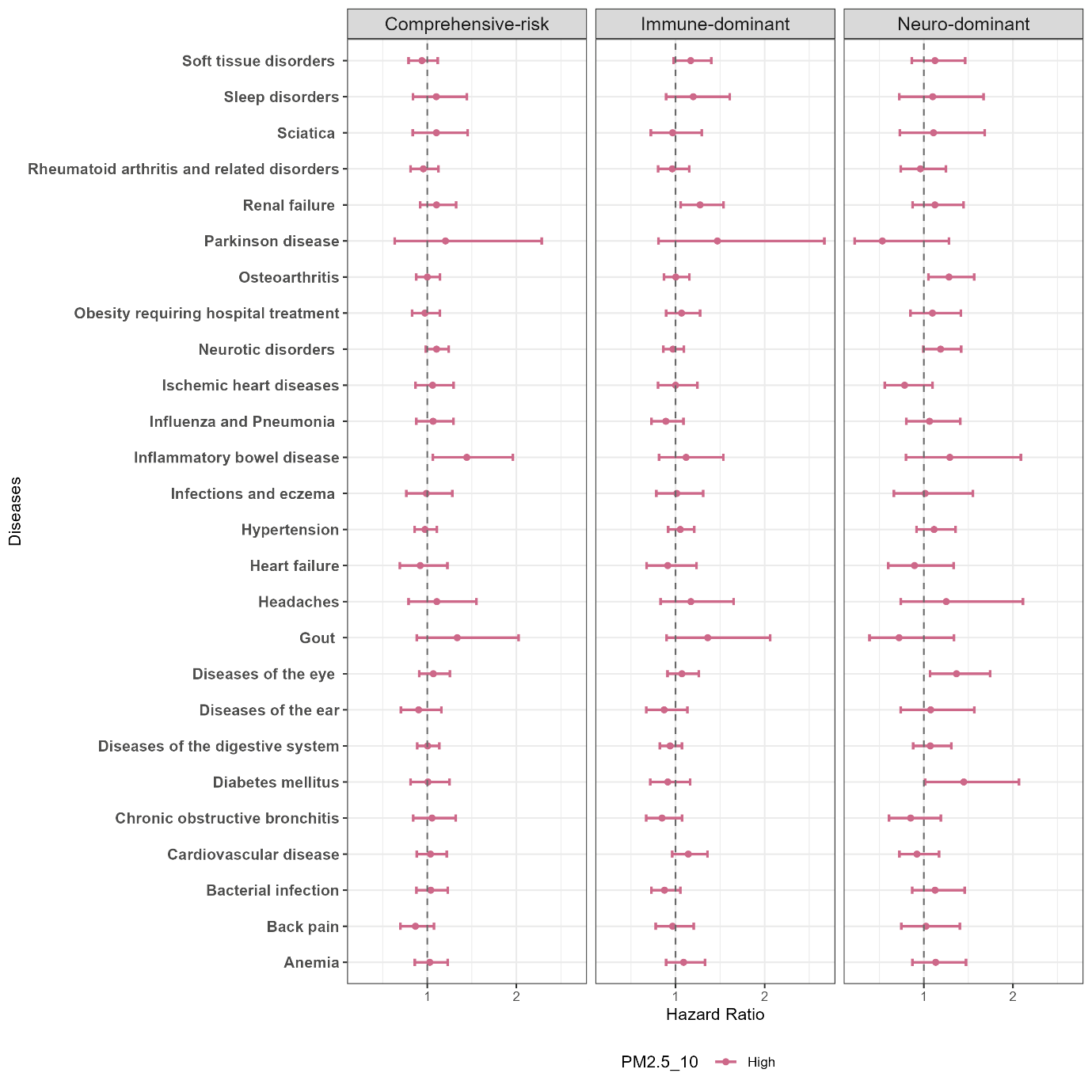
**

**Figure S6**. Association between PM_2.5-10_ exposure and secondary diseases of depression across different genetic subtypes.

***** The x-axis represents the hazard ratio (HR), with points and error bars indicating the HR and 95% confidence intervals (CI). The low PM_2.5-10_ exposure was considered reference.

**
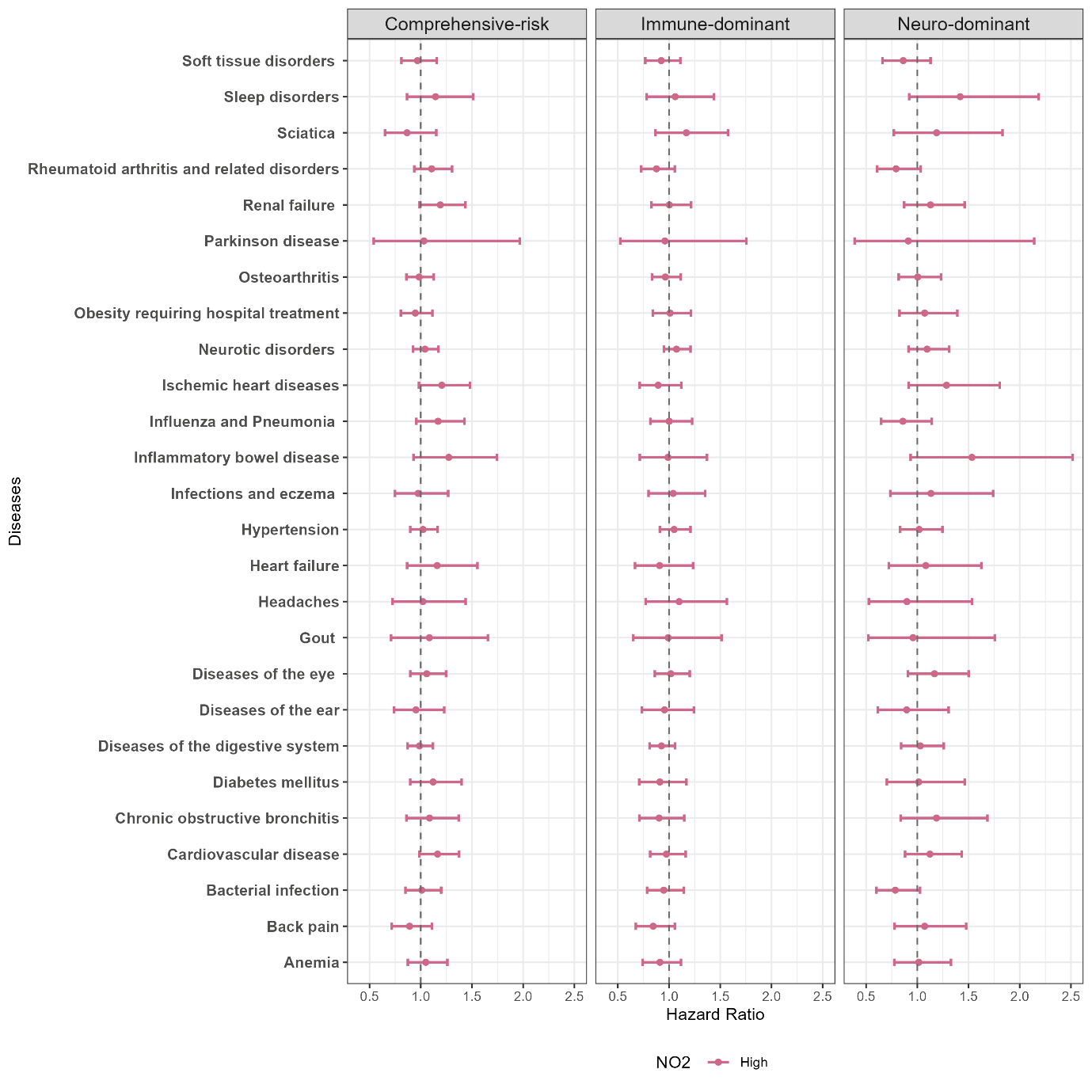
**

**Figure S7**. Association between NO_2_ exposure and secondary diseases of depression across different genetic subtypes.

***** The x-axis represents the hazard ratio (HR), with points and error bars indicating the HR and 95% confidence intervals (CI). The low NO_2_ exposure was considered reference.

**
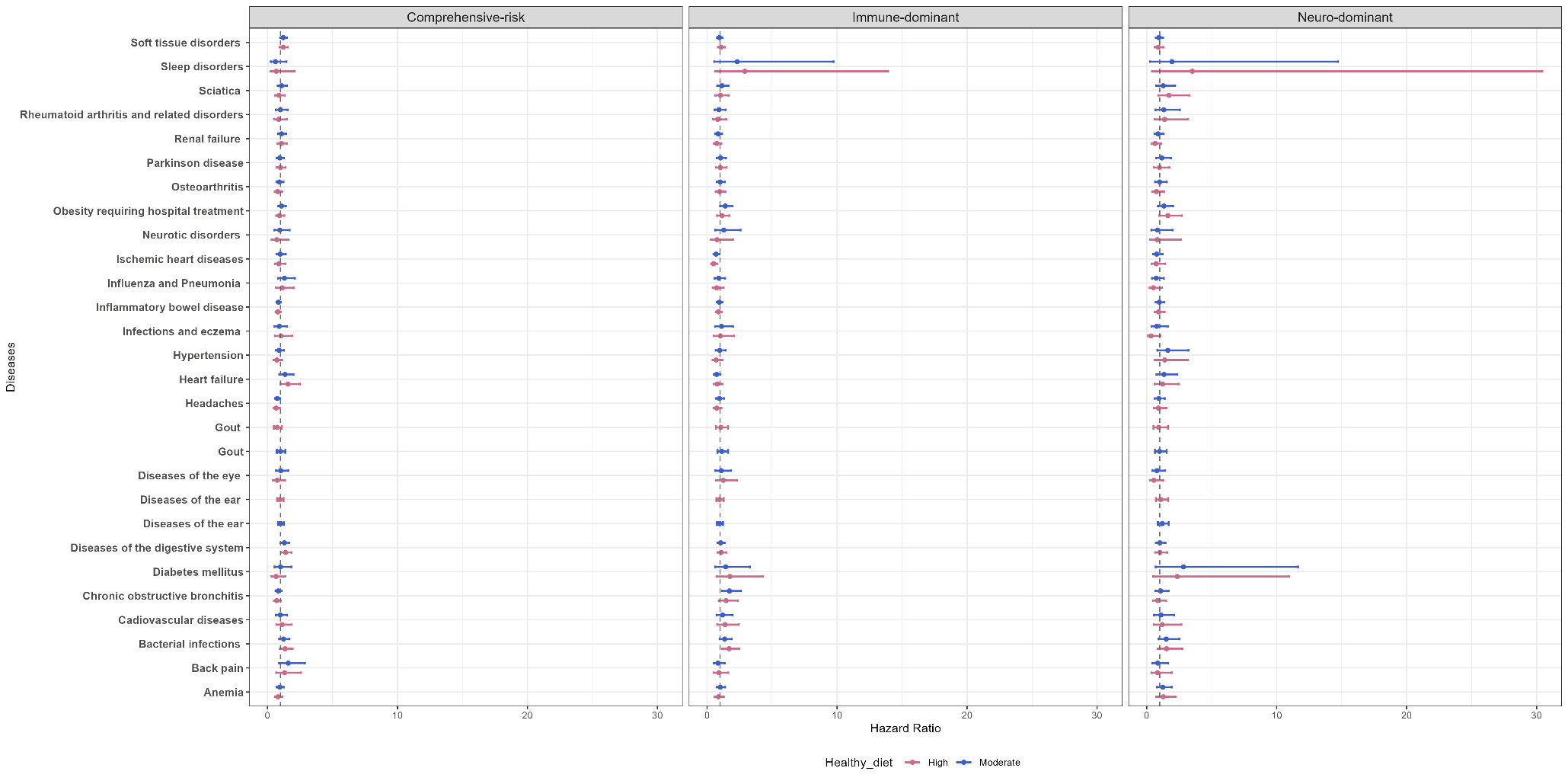
**

**Figure S8**. Association between healthy diet and secondary diseases of depression across different genetic subtypes.

***** The x-axis represents the hazard ratio (HR), with points and error bars indicating the HR and 95% confidence intervals (CI). The low healthy diet was considered reference.
